# Subjective and Perseverative Cognition Mediate the Relationship Between Sleep and Work Impairment

**DOI:** 10.1093/geroni/igab046.434

**Published:** 2021-12-17

**Authors:** Christina Mu, Brent Small, Soomi Lee

**Affiliations:** University of South Florida, Tampa, Florida, United States

## Abstract

The study examined the mediating role of subjective and perseverative cognition on sleep and work impairment. Sixty nurses completed a background survey and 14-days of ecological momentary assessments (EMA) and sleep actigraphy. Each day, participants evaluated their subjective cognition (mental sharpness, memory, processing speed), perseverative cognition (rumination) and work impairment (how much did you cut back on normal paid work, how much did the quality of your work suffer). Multiple sleep characteristics were measured by EMA and actigraphy. Multilevel mediation models adjusted for sociodemographics and work shift. At the between-person and within-person levels, there were mediated associations of sleep quality and sufficiency (but not actigraphy-measured sleep) with work impairment through subjective and perseverative cognition. Better sleep quality or higher sleep sufficiency were associated with better subjective and perseverative cognition, which, in turn, were associated with less work impairment.

